# Short‐chain fatty acids profile in patients with SARS‐CoV‐2: A case‐control study

**DOI:** 10.1002/hsr2.1411

**Published:** 2023-07-06

**Authors:** Edris Nabizadeh, Mohammad Yousef Memar, Hamed Hamishehkar, Hadi Ghanbari, Hiva Kadkhoda, Solmaz Asnaashari, Hossein Samadi Kafil, Mojtaba Varshochi, Mostafa Mostafazadeh, Rasoul Hosseinpour, Reza Ghotaslou

**Affiliations:** ^1^ Student Research Committee Tabriz University of Medical Sciences Tabriz Iran; ^2^ Infectious and Tropical Diseases Research Center Tabriz University of Medical Sciences Tabriz Iran; ^3^ Drug Applied Research Center Tabriz University of Medical Sciences Tabriz Iran; ^4^ Department of Pharmacognosy, Faculty of Pharmacy Tabriz University of Medical Sciences Tabriz Iran; ^5^ Biotechnology Research Center Tabriz University of Medical Sciences Tabriz Iran; ^6^ Department of Biochemistry and Clinical Laboratories Tabriz University of Medical Sciences Tabriz Iran

**Keywords:** acetic acid, butyric acid, propionic acid, SARS‐CoV‐2, short‐chain fatty acids

## Abstract

**Background and Aims:**

SARS‐CoV‐2, as a new pandemic disease, affected the world. Short‐chain fatty acids (SCFAs) such as acetic, propionic, and butyric acids are the main metabolites of human gut microbiota. The positive effects of SCFAs have been shown in infections caused by respiratory syncytial virus, adenovirus, influenza, and rhinovirus. Therefore, this study aimed to evaluate the concentration of SCFAs in patients with SARS‐CoV‐2 compared with the healthy group.

**Methods:**

This research was designed based on a case and control study. Twenty healthy individuals as the control group and 20 persons admitted to the hospital with a positive test of coronavirus disease (COVID‐19) real‐time polymerase chain reaction were included in the study as the patient group from September 2021 to October 2021, in Tabriz, Iran. Stool specimens were collected from volunteers, and analysis of SCFAs was carried out by a high‐performance liquid chromatography system.

**Results:**

The amount of acetic acid in the healthy group was 67.88 ± 23.09 μmol/g, while in the group of patients with COVID‐19 was 37.04 ± 13.29 μmol/g. Therefore, the concentration of acetic acid in the patient group was significantly (*p* < 0.001) lower than in the healthy group. Propionic and butyric acid were present in a higher amount in the control group compared with the case group; however, this value was not statistically significant (*p* > 0.05).

**Conclusion:**

This study showed that the concentration of acetic acid as the metabolite caused by gut microbiota is significantly disturbed in patients with COVID‐19. Therefore, therapeutic interventions based on gut microbiota metabolites in future research may be effective against COVID‐19.

## 1 INTRODUCTION

Coronavirus disease (COVID‐19) was first reported from Wuhan, China in December 2019, and this infectious and highly contagious agent led to a pandemic of acute respiratory syndrome. Research based on molecular methods confirmed the origin of this virus as a new SARS‐CoV‐2.^1^ SARS‐CoV‐2 is mainly transmitted through the respiratory droplets created by infected persons. Despite widespread vaccination against this viral disease, many countries of the world are still involved. Researchers also claim that this virus may be with us forever. The virus in the respiratory system to enter into cells attaches to the angiotensin I converting enzyme 2 (ACE‐2), as its specific receptor site, then this action is terminated with the help of transmembrane protease serine 2.^2^ In addition to the respiratory tract, the ACE‐2 receptor is also widely expressed in the gastrointestinal (GI) tract.^3^ Therefore, the infection of the COVID‐19 disease is not only limited to the respiratory system. A systematic meta‐analysis study showed that 20% of people also suffer from GI problems.^4^ Since SARS‐CoV‐2 is detected in the stool sample of 50% of the involved people, it makes the intestine a suitable place for the replication of this virus and a source for its transmission.^5^, ^6^ On the other hand, gut microbiota, which is considered a forgotten organ of the body, has undeniable importance on human health. The activity of stimulating and improving the immune system by gut microbiota highlights the importance of this new organ, especially against viral diseases. In this process, metabolites resulting from the activity of gut microbiota members play a prominent role.

Short‐chain fatty acids (SCFAs) such as acetic acid, propionic acid, and butyric acid are the main metabolites of gut microbiota. Acetate is higher concentrations than propionate and butyrate in the intestine, and the concentration of propionate and butyrate are almost equal.^7^ The impact of SCFAs compounds in the intestine are including resource of energy production for colonocytes, anti‐inflammatory role, and regulation of immune system responses.^8^ The remarkable point is that the effects of these compounds are not only limited to the intestines and digestive system. From this perspective, SCFAs can affect other parts of the body, such as the lungs by entering the bloodstream.^9^ Several studies have shown the protective effect of SCFAs against infectious agents. The mechanism of action can be directly on the infectious agent or indirectly through the immune system.^10^, ^11^ In previous studies, the positive effects of SCFAs have been shown for infections caused by respiratory syncytial virus (RSV), adenovirus, influenza, and rhinovirus.^12^, ^13^ Meanwhile, the relationship between SCFAs and COVID‐19 disease is less understood. Therefore, this study aimed to evaluate the concentration of SCFAs in patients with SARS‐CoV‐2 compared with the healthy group.

## 2 MATERIALS AND METHODS

### 2.1 Study design and sample selection

This research was designed based on a case and control study. For this purpose, 20 healthy individuals as the control group, and 20 persons admitted to the hospital with a positive real‐time polymerase chain reaction test of COVID‐19 were included in the study as a case group from September 2021 to October 2021, in Tabriz, Iran. After matching the age and gender, healthy volunteers were selected as the control group. The exclusion criteria for both groups were taking any antibiotics and probiotics for at least 2 months before sampling. Stool samples were collected and immediately stored at −80°C until to do the test.

### 2.2 Fecal SCFAs extraction

SCFA extraction and high‐performance liquid chromatography (HPLC) analysis were performed based on a previous study with some changes.^14^ At first, 300 mg stool sample was transferred to a 2 mL microtube and mixed with 1 mL of deionized distilled water, and centrifuged at 12,000 rpm for 10 min. Then, 100 μL of absolute HCl was added to the supernatant solution. Also, 5 mL of diethyl ether was added to this solution and placed on the rotator for 20 min, and centrifugation was performed for 5 min at 3500 rpm. After that, 500 μL of NaOH 1 N was added to the organic phase. The centrifugation was carried out for 5 min at 3500 rpm again, and 100 μL of absolute HCl was mixed with the sediment and was vortexed. Finally, after filtration, this solution was ready to be injected into the HPLC system.

### 2.3 High‐performance liquid chromatography

Analysis of SCFAs was carried out by a Smart line Knauer HPLC system containing a pump (Smartline pump 1000, Knauer), a ultraviolet (UV) detector (Smart line UV detector 2600, Knauer), and a 20‐μL Knauer injection loop. Identification of SCFAs was performed at a wavelength of 210 nm. The chromatographic patterns were monitored by the EZChrom Elite system (Knauer), and Chromate software was used for processing data. A C18 column (250 mm × 4.6 mm ID, 5 µm) (Knauer) was used for analytes separation. A solution containing 95% sodium dihydrogen phosphate (NaH_2_PO_4_) 20 mM with a pH adjusted to 2.5, and 5% of acetonitrile after filtration and sonication were used as the mobile phase. The standards of acetic, propionic, and butyric acid were purchased to identify and evaluate their concentration in the stool samples of patients with COVID and healthy individuals.

### 2.4 Statistical analysis

Data analyses were carried out using GraphPad software 9.4.1. Descriptive statistics were conducted to assess the demographic and clinical variables. Analytical statistics were used to compare the mean concentration of SCFAs in two groups. For this purpose, the data were evaluated for normality using the Shapiro–Wilk and Kolmogorov–Smirnov tests. For normal data, the student's *t* test was used to compare the mean in two groups, and Mann–Whitney *U* test was used for nonnormal data. Quantitative values were reported based on the mean ± standard deviation, and a *p* < 0.05% was considered for significant results.

## 3 RESULTS

Table 1 shows some demographic information of the patient group and healthy group. As shown, there is no significant difference concerning age and gender variables in the two groups. The mean age of the patient group was 55.40 ± 13.21 years, and the healthy group was 54 ± 12.55 years old.

**Table 1:** Some demographic information of the participants in this study.

Variables	Patients group (*n* = 20)	Healthy group (*n* = 20)	*p* Value
Age (Y) (mean ± SD)	55.40 ± 13.21	54 ± 12.55	0.733
Gender (male/female)	13/7	13/7	1.000

Abbreviations: Y, years; SD, standard deviation.

**Figure 1:**
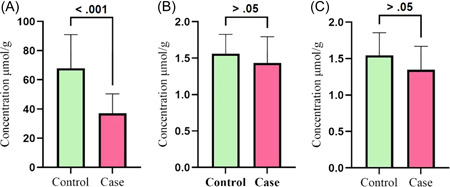
Concentration of short‐chain fatty acids in healthy volunteers and patient group. (A) Acetic acid, (B) propionic acid, and (C) butyric acid.

In general, in both groups, the concentration of acetic acid was higher than propionic and butyric acid. The amount of acetic acid in the healthy group was 67.88 ± 23.09 μmol/g, while in the group of patients with COVID‐19, this value was 37.04 ± 13.29 μmol/g. Therefore, the concentration of acetic acid in the patient group was significantly lower than in the healthy group (*p* < 0.001, Figure 1A). Propionic acid was observed in the healthy group with a concentration of 1.56 ± 0.26 μmol/g, and in the patient group, this amount was 1.43 ± 0.35 μmol/g. The proportion of propionic acid in the healthy group was higher than that in the patient group, but this value was not statistically significant (*p* > 0.05, Figure 1B). The amount of butyric acid in the healthy group was 1.54 ± 0.30 μmol/g, and that in the patients with COVID‐19 was 1.34 ± 0.32 μmol/g. Similar to propionic acid, the concentration of butyric acid was higher in the healthy group, but there was not a statistically significant relationship (*p* > 0.05, Figure 1C).

## 4 DISCUSSION

In recent years, it became clear that the impact of gut microbiota on human health is undeniable.^15^ This function can be directly related to the presence of microbiota members in the intestine or the metabolites produced by these members. SCFAs such as acetate, propionate, and butyrate are the main metabolites produced by the intestinal microbiota. These compounds, directly and indirectly, affect the host health by improving the protective and immune mechanisms. The noteworthy point is that the function of SCFAs is not limited to the GI tract. These metabolites can enter the bloodstream and affect all parts of the body, especially the lungs. On the other hand, 2 years after the COVID‐19 pandemic and widespread vaccination, the world population is still involved with this virus. However, different finding strategies to overcome this situation can be effective. Therefore, this study was designed to evaluate the concentration of SCFAs in patients with COVID‐19 disease and healthy volunteers.

Our results showed significant changes in the concentration of SCFAs between the two groups (Figure 1). In both groups, the concentration of acetic acid was much higher than propionic and butyric acid. Acetic acid has two carbon atoms and is the shortest fatty acid in the intestine. The effects of energy production, maintaining body homeostasis, improving the immune system, and the anti‐inflammatory role of acetic acid have been shown in various studies.^16^ Furthermore, various studies have evaluated the antiviral effects of acetate. Studies have shown that acetate can reduce the severity of RSV infection by regulating retinoic acid‐inducible gene‐I expression and some affect related to IFN‐, GPR43, and IFNAR.^17^ However, with a similar mechanism, it was found that acetate affects SARS‐CoV‐2 infection by reducing the expression of the ACE‐2 receptor. In agreement with these findings, our study showed a significant decrease in acetate concentration in patients with COVID‐19. Therefore, according to the previous findings, it can be concluded that acetate can play a role in the pathophysiology of COVID‐19 disease.

Propionate and butyrate are other important SCFAs with three and four carbon atoms, respectively. Studies have focused more on propionate and butyrate than other SCFAs. These two SCFAs affect cell signaling pathways by two mechanisms. First, propionate and butyrate have an inhibitory role in gene expression by affecting histone decarboxylase.^18^ Second, these metabolites lead to signal transmission from the G protein‐coupled receptors route.^19^ This activity leads to the expression of GPR109A and GPR43 on macrophages, neutrophils, and dendritic cells, and the consequences of this action ultimately improve the function of immune system cells.^20^ The main challenge in SARS‐CoV‐2 infections is the overexpression of ACE‐2 receptors, which is influenced by factors such as diabetes, cardiovascular diseases, and smoking.

Evidence shows that propionate and butyrate lead to a decrease in the expression of ACE‐2 receptor through the effect on cell signaling.^21^, ^22^ Beyond this effect, Takabayashi et al. showed that propionate and butyrate with other SCFAs even lead to a reduction in SARS‐CoV‐2 genome expression.^23^ However, contrary to these results, our study showed that although the concentration of propionate and butyrate was higher in the healthy group, the difference was not statistically significant. Therefore, the findings related to propionate and butyrate in this study were not in agreement with the previous investigation. Among the main reasons for this difference, we can point to factors such as microbiota diversity, type of diet, and host genotype.^24^ But in agreement with our findings, a study showed that propionate and butyrate do not play a role in the pathophysiology of SARS‐CoV‐2 infection.^25^ Most of the studies indicate the positive role of SCFAs on the disease of COVID‐19; however, some studies have not shown this effect. The relationship between SCFAs and human health is not well understood. So, the design of more extensive studies in this field is suggested.

As a limitation of this study, we were not able to evaluate other metabolites of the gut microbiota. In our study, acetate concentration was significantly lower in the patient group. This result can be more prominent and confirm the effect of acetate on human health by evaluating it on laboratory animals.

## 5 CONCLUSION

This study showed that the concentration of acetic acid as the metabolite caused by intestinal microbiota is significantly disturbed in patients with COVID‐19. However, no significant relationship was observed in the concentration of propionic acid and butyric acid in the two groups, although this value was lower in the patient group. Therefore, therapeutic interventions based on gut microbiota metabolites in future research may be effective in controlling the disease of COVID‐19.

## AUTHOR CONTRIBUTIONS

**Edris Nabizadeh**: Conceptualization; methodology; project administration; writing—original draft; writing—review and editing. **Mohammad Yousef Memar**: Formal analysis; software; writing—review and editing. **Hamed Hamishehkar**: Methodology; software; writing—review and editing. **Hadi Ghanbari**: Formal analysis; software; writing—review and editing. **Hiva Kadkhoda**: Writing—original draft; writing—review and editing. **Solmaz Asnaashari**: Methodology; validation; writing—review and editing. **Hossein Samadi Kafil**: Investigation; writing—review and editing. **Mojtaba Varshochi**: Supervision; writing—review and editing. **Mostafa Mostafazadeh**: Formal analysis; software; writing—review and editing. **Rasoul Hosseinpour**: Data curation; writing—review and editing. **Reza Ghotaslou**: Conceptualization; project administration; writing—original draft; writing—review and editing.

## CONFLICT OF INTEREST STATEMENT

The authors declare no conflict of interest.

## ETHICS STATEMENT

All volunteers who entered this study signed the informed consent form approved by the Ethics Committee of Tabriz University of Medical Sciences (Ref. No. IR.TBZMED.REC.1400.538).

## TRANSPARENCY STATEMENT

The lead author Reza Ghotaslou affirms that this manuscript is an honest, accurate, and transparent account of the study being reported; that no important aspects of the study have been omitted; and that any discrepancies from the study as planned (and, if relevant, registered) have been explained.

## Data Availability

The data that support the findings of this study are available from the corresponding author upon reasonable request.
